# Religion and HIV-Related Stigma among Nurses Who Work with People Living with HIV/AIDS in Puerto Rico

**DOI:** 10.1177/2325958218773365

**Published:** 2018-05-14

**Authors:** Marcos Reyes-Estrada, Nelson Varas-Díaz, Richard Parker, Mark Padilla, Sheilla Rodríguez-Madera

**Affiliations:** 1 School of Behavioral and Brain Sciences in Ponce, Ponce Health Sciences University, Ponce, Puerto Rico; 2 Department of Global and Sociocultural Studies, Florida International University, Miami, FL, USA; 3 Mailman School of Public Health, Columbia University, New York, NY, USA; 4 Department of Social Sciences, Medical Sciences Campus, University of Puerto Rico, San Juan, Puerto Rico

**Keywords:** religion, HIV/AIDS, nursing, stigma, Latinos

## Abstract

HIV-related stigma among nurses can impact health care services for people with HIV/AIDS (PWHA). health care professionals’ religious views can potentially foster stigmatizing attitudes. There is scarce scientific literature exploring the role of religion on HIV/AIDS stigma among nurses. This study aimed to explore the role of religion in the stigmatization of PWHA by nurses in Puerto Rico. We conducted an exploratory study using qualitative techniques. We conducted 40 in-depth interviews with nurses who provided services to PWHA. Three main factors emerged in the analysis as contributors to HIV/AIDS stigmatization: (1) nurses’ personal religious experiences, (2) religion as a rationale for HIV-related stigma, and (3) religious practices during health care delivery. The results show that religious beliefs play a role in how nurses understood HIV/AIDS and provided service. Results point toward the need for interventions that address personal religious beliefs while reducing HIV/AIDS stigma among nurses.

There are an estimated 1.1 million people with HIV/AIDS (PWHA) in the United States.^1^ In 2016, more than 39 000 individuals were diagnosed with HIV. HIV has disproportionately affected minority populations, such as gay and bisexual men, African Americans, and Hispanics/Latinos. For example, young Hispanics/Latinos and Black/African Americans between 13 and 24 years of age accounted for 79% of new diagnoses in 2016 among those who reported unprotected sexual intercourse between men.^1^ African Americans and Hispanics/Latinos represent the 2 highest incidence rates of HIV infection when ranked by race and ethnicity. It is estimated that 83% of new diagnoses among men are among gay and bisexual men.^1^ HIV continues to disproportionally affect racial/ethnic minority populations, as well as stigmatized populations (eg, men who have sex with men [MSM] and people who inject drugs).

In Puerto Rico, there are more than 47 000 cumulative HIV/AIDS diagnoses.^2^ A total of 43% of all HIV infections is attributed to transmission via injection drug use (IDU), 29% to unprotected heterosexual intercourse, and 19% to unprotected sexual intercourse among MSM. This scenario highlights the disproportionate burden of HIV infection among IDU and MSM in Puerto Rico. Moreover, HIV infection among MSM has continually increased in recent years.^2^ Given their linkage to the populations most vulnerable to HIV/AIDS, stigma and discrimination are among the foremost barriers to HIV prevention, treatment, care, and support in Puerto Rico.^3^

## HIV-Related Stigma and Nurses

HIV-related stigma should be understood as a social process based on an unbalanced hierarchical relationship of power. This understanding was informed by Goffman’s^4^ work on social stigma, in which he described stigma as “an attribute that is significantly discrediting” to the reputation of the stigmatized individual.^4^ The context of social relationships is central to Goffman’s notion of stigma.^5^ It is within the relationship context that individuals are labeled or defined as deviant beings. This labeling process takes place in different settings including among health care workers who care for PWHA, such as nurses.

Since the beginning of the HIV epidemic, nurses have played a crucial role in effectively providing and managing HIV treatment. Nurses are in an important position to advocate and deliver high-quality and effective treatment to PWHA. Nurses’ critical role in HIV treatment includes providing prevention information, counseling, support, and prompt linkage to health care services within the HIV treatment cascade.^6^ However, a body of research has documented HIV-related stigma manifestations that can hinder nurses’ delivery of care. For example, researchers have documented negative attitudes toward PWHA, lack of knowledge related to HIV transmission, fear of contagion, and social stigma.^7-12^ Research has shown that stigma manifestations among professional nurses can be manifested by behaviors such as refusing/avoiding to collect blood specimens and wearing more gloves and masks than needed because of fear of infection.^12,13^

Stigma manifestations have been a public health concern since the onset of the HIV/AIDS epidemic. Studies have documented the detrimental effects of HIV/AIDS-related stigma on prevention strategies, patients’ adherence to treatment, and psychological and physical well-being of PWHA, among other factors.^14-16^ This scenario is more complex when HIV/AIDS stigma is manifested by professional nurses, who are in a particularly critical position in which to provide support and link patients to HIV-related health care services.^6^ Scientific literature on HIV/AIDS-related stigma has emphasized cognitive, individual, and interpersonal factors that are relevant to understanding the stigmatization process among health care professionals (eg, a health care professional’s accurate knowledge of how the virus works, how to manage effectively his/her emotions during clinical interactions, and the degree of proximity to the stigmatized group).^17,18^ Moreover, researchers have called for an examination of the social structural factors underlying stigma, and religion has consequently emerged as a social phenomenon that can foster or contribute to stigmatization.^20,21^

## Culture, Religion, and HIV-Related Stigma

The devaluation of stigmatized individuals does not occur in a vacuum, but in a larger context of intersecting systems and hierarchical power dynamics.^22^ Thus, the intersection between “culture,” “power,” and “differences” are pivotal to understanding the contexts in which stigmatization occurs and the social structures that shape stigma manifestations. Within this framework, religious communities can foster moral judgments about PWHA by promoting negative attitudes toward the disease and the communities most affected.^22,23^ These negative attitudes, which may be expressed unevenly within a religious community, echo the sentiment of the early years of the epidemic in the United States when the disease was routinely associated with sinful behaviors believed to compromise the “social order.”^19,21,24^ Today, religious communities have evolved greatly in their work on HIV/AIDS, and yet the religion–moral justifications for the stigmatization of PWHA continue to circulate in many societies, including Puerto Rico.

Religion is a cultural and psychosocial phenomenon of large proportions in the United State and Puerto Rico. In 2014, the Pew Research Center^25^ conducted the US Religious Landscape Survey with a representative sample and showed that 70.6% of adults surveyed self-identified as Christian. In addition, for racial and ethnic minorities, Catholicism represented 41%, Evangelical Protestantism 24%, and Protestantism 14%. In this context, it is important to note that Puerto Rico’s colonial history has shaped its religious landscape. For example, on the island, most of the population self-identifies as Roman Catholic (56%) and as Protestant (33%).^26^

In Puerto Rico, scientific research related to HIV stigma among professional nurses has been scarce.^27^ However, some research has documented HIV-related stigma manifestations among health care professionals, including samples of Puerto Rican nursing students, which highlight that those nursing students who reported participating in religious activities scored higher on levels of HIV-related stigma than those who did not.^28^ Furthermore, upon detailed examination of the stigma measurement scale’s subdimensions, the researchers found that those who participated in religious events scored significantly higher on 4 of the 11 dimensions of the stigma scale. Specifically, these participants scored higher on the following aspects: (1) interpreting PWHA as less productive, (2) believing that personal characteristics such as irresponsibility caused HIV infection, (3) being fearful of becoming infected in everyday social interaction, and (4) having more negative emotions associated with PWHA (eg, shame, pity, and anger).^28^ However, it is important to note that this study only included a small sample of nursing students and participants did not have experience working directly with PWHA at the moment of the study. This highlights the need to explore the role of religion and HIV-related stigma among Puerto Rican nurses who are working directly with PWHA.

A growing body of research has highlighted how religious beliefs can have a significant effect on HIV-related stigma manifestations.^21^ For example, Andrewin and Chein^20^ reported HIV-related stigma among health care professionals, including nurses, and found that those professional who identified as religious were more stigmatizing, in particular with blaming and judging PWHA. Transmission of HIV in the context of drug use, same-sex behavior, and having multiple sex partners has been associated with sinful behaviors.^13,21^ Indeed, “sin” seems to underlie the stigma toward PWHA in these studies. However, the scenario is more complex considering how both religion and health care services have been associated with positive-health outcomes in other research. Researchers have documented how religious factors could foster improvement in quality of health and coping skills among PWHA.^29,30^ Other authors have also suggested a correlation between religiosity and adherence to highly active antiretroviral therapy,^31^ which would have crucial implications for health outcomes among PWHA. Thus, a critical approach to the study of religion and HIV-related stigma must take into consideration its dual role: a potentially positive effect on PWHA’s quality of life and the capacity of religion to foster stigmatization by health care providers (among others).

Although research has highlighted the important role of religion in HIV-related stigmatization by health professionals,^20^ it has less frequently addressed it among nurses. Considering that Puerto Ricans place a great deal of importance on religion and that nurses play a fundamental role in HIV/AIDS care on the island, it is imperative to have a better understanding of how religion influences HIV-related stigma among nurses. Consequently, this study aimed to explore the role of religious beliefs in the processes of stigmatization toward PWHA among Puerto Rican nurses.

## Methods

To achieve the proposed aim of the study, we implemented an exploratory design using qualitative techniques consisting of in-depth semi-structured interviews with Puerto Rican nurses who worked with PWHA at the time of data collection.

### Participants

The sample consisted of 40 Puerto Rican nurses who provided services to PWHA on the island. The participating nurses met the following criteria: (1) being older than 21 years of age, (2) living in Puerto Rico at the time of their interview, (3) voluntarily participating in the study, (4) being active practitioners of their profession at the moment of the interview, and (5) working in a health institution in which PWHA received services (eg, clinics specialized in HIV treatment). The sample consisted mostly of women (82%). Regarding their professional expertise in the area of HIV/AIDS, 85% had specialized training in HIV care and 48% provided services in HIV clinics. Most participants (93%) reported that their religious beliefs were “important” or “very important” in their lives. Catholics were the most prominent (33%) religious group among the sample (Table 1).

**Table 1.** Frequency Distribution of the Participants in Sociodemographic Variables.

Variables	Frequency	Percent
Gender		
Male	7	18
Female	33	82
Age		
24-34 years	7	18
35-45 years	7	18
46-56 years	19	48
57-67 years	6	15
67 years or more	1	3
HIV/AIDS training		
Yes	34	85
No	6	15
Religious group		
Catholic	13	33
Protestant	11	28
Evangelic	9	23
Adventism	4	10
Jehovah’s Witness	1	3
Episcopal	1	3
Importance of religion		
Nothing	1	5
Somewhat	2	5
Important	7	18
Very important	30	75
Participation in religious activities (eg, church)		
Never	3	8
Once in a year	4	10
Monthly	6	15
Weekly	22	55
Daily	5	12
Participation in private activities (eg, pray)		
Never	0	0
Once in a year	0	0
Monthly	4	10
Weekly	2	5
Daily	34	85

^a^N = 40.

### Procedure

The study’s protocol was evaluated and approved by the Institutional Review Board for the Protection of Human Subject at the Ponce Health Sciences University. Participants were contacted via the Puerto Rican Nurse Association, which is the main professional organization of nurses on the island. We also used promotional materials (ie, brochures) for recruitment. We explained the nature of the study, and for those who agreed to participate, we proceeded to coordinate the in-depth interview. Interviews took place in settings that were private, with no interruptions and were participants felt comfortable. Once participants read and signed the informed consent forms, we applied a demographic questionnaire and then conducted the interviews, which were audio-recorded for transcription, qualitative coding, and analysis. The interviews were completed in Spanish, and participants received a stipend of US$20.00 to cover transportation costs.

### Instruments

We developed 2 instruments to gather data, which were reviewed by a panel of experts in qualitative methodology and research on HIV and stigma. The first one was the *Socio-demographic Data Questionnaire* that included questions related to gender, age, income, years in the nursing profession, expertise in HIV, and religious beliefs, among others. The second one was the *In-depth Semi-Structured Interview Guide* that was used to provide some level of uniformity during the interview process. This guide included questions related to the following areas: (1) perceptions about the HIV epidemic in Puerto Rico, (2) nurses’ professional contributions to the HIV epidemic, (3) religious beliefs, (4) the role of religion in HIV service provision, and (5) contributions of religious organizations to the HIV epidemic. It also included 3 vignettes on HIV-related cases that were used to elicit attitudes toward PWHA.^32^ These 3 vignettes addressed the following cases: (1) a man with HIV infected via unclean needle sharing during illegal drug use, (2) a man with HIV that disclosed his religious beliefs and anger toward God during the clinical encounter, and (3) and a woman infected via unprotected heterosexual intercourse with her husband.

### Analysis

Before the transcription and analysis processes, members of the research team were trained on data quality procedures and analysis by an expert in qualitative research. After several trainings sessions, the research team began the interview and transcription process. After the interviews were transcribed, the research team met on a weekly basis to identify themes and patterns that emerged from the transcriptions. During these meetings, the research team reflected on the aims of the study while reviewing transcripts conjointly to identify emergent themes providing insight into religious beliefs, religious practices, work experiences, and stigmatization toward PWHA. Subsequently, the research team developed a master list of themes for a focused coding analysis. The master list was a well-described but flexible guide in which themes could be added as the analysis proceeded but which provided direction for the team. Once the general themes and patterns were identified in all interviews, the research team searched for excerpts from the texts that best exemplified them. Selected passages from the texts on which all 3 analysts agreed were included in the final interpretation of meta-themes related to the stigmatization of PWHA by nurses. For those sections of texts in which analysts disagreed regarding their expression of a theme, we engaged in discussion to either reach consensus or develop more nuanced categories to account for our variations. These steps were carried out to ensure that the analysts agreed on the final interpretation of the coded passages and to avoid the inclusion of verbalizations that are unclear in their meaning or expression of stigma.^33^ Finally, once the texts were appropriately selected and discussed by the research team, they were coded with the use of qualitative analysis computer software (ie, NVivo (QSR International) V.9).^34^ Two experts on HIV-related stigma and qualitative research, who were fluent in both Spanish and English, guided the translation of the selected text in order to ensure the appropriate translation of the passages.

## Results

We identified 3 main thematic categories that evidenced the potential intersection between religion and HIV-related stigma manifestations. In this section, we present text excerpts related to 3 thematic categories: (1) nurses’ personal religious experiences, (2) religion and HIV-related stigma, and (3) religious practices during health care delivery (Table 2).

**Table 2.** Main Categories of Analysis.

Category	Description
Nurses’ personal religious experiences	Verbalizations related to personal religious beliefs and practice among participants. These included organizational and individual practices in which religion was the main driving factor.
Religious practices during health care delivery	Verbalizations related to the use of religion during service provision to PLWHA.
Religion as a rationale for HIV-related stigma	Stigmatizing verbalizations related to PLWHA. These included using personal characteristics to explain HIV infection, descriptions of denial of services due to religion, and conceptualizing HIV infection as the consequence of breaking religious laws.

Abbreviations: PLWHA, people living with HIV/AIDS.

### Nurses’ Personal Religious Experiences

In this category, we included verbalizations where participants self-identified as religious persons and described their religious experiences in their everyday life. All participants reported having some sort of religious beliefs and engaging in religious activities. For example, one nurse explained it in the following manner:

Interviewer (I): How does religion impact your daily life (…)?

Participant (P): I have always believed in God. I have always had my beliefs that God exists and is part of my life. I go to church 3 times per week, and I emphasize religion in my life and try to guide people through the same way.

Another participant described his religious beliefs and how intrinsic these were to his daily life:

I: Do you consider yourself a religious person?

P: Yes…I may not have a nun’s habit, but yes!

I: How does religion impact your daily life (…)?

P: For me, it is something normal in my life. I get up in the morning. I drive my car. I pray…sometimes at noon, or during the morning.

Finally, one participant provided a more detailed description of the role of religion in her life, stemming from childhood. She narrated her adulthood as a period where she felt discomfort with her Catholic faith and searched for a new church. The following excerpt describes the importance of religion in her life:

I: How does religion impact your daily life?

P: I’ll tell you, I was raised in the Catholic church. I participated in the Church choir; then I started going to the university and I felt an emptiness. I knew that the Catholic Church was not for me. I left the Church and then I visited an Evangelical Church with a friend. I liked it because they taught you that you cannot judge others. I can tell you that both, professionally and personally, it has helped me. Maybe there were members of that Church who judged people, but I never saw the pastor and his wife judging. At least, I never saw anything like that.

I: And does that make you feel good?

P: Yes, it made me feel good with myself and I think with the others. I don’t think I have problems with anyone. The Church helped me a lot, I can tell you that, and so much in the spiritual area. There are people who don’t believe in anything, but I respect your decisions and beliefs. I am currently not active in any Church. I am looking for one, but my roots are already there.

I: And you told me at the beginning of the interview that you continue with individual religious practices.

P: Yes, I read the Bible every day. I read a lot of Christian things. I try to include and educate my daughters with positive things. Not to be prejudiced…they know that.

Participants’ verbalizations throughout the study evidenced that religion was an important aspect of their daily lives manifested through their engagement in formal religious activities and personal practices (eg, praying). Although some participants recognized that religious individuals can hold negative attitudes toward others, religion was mainly described as a strategy to hold positive relations with different people and socialize children into a prejudice-free worldview, as the above example demonstrates. Nurses also reported bringing their religious beliefs and experiences into the health care settings in which they worked, as we describe in the next section.

### Religious Practices during health care Delivery

In this category, we included narratives where participants described implementing personal religious practices during health care delivery. Participants understood that taking care of PWHA was an important part of their religious beliefs and practices. In addition, they considered that the provision of nursing services to PWHA was evidence of having an intrinsic relationship with God. One nurse mentioned the following:

I: How does religion impact your clinical practice?

P: I’ve always wanted to serve the patient, regardless of the clinic. I feel that it is a human commitment and all this comes from the religious background that one has. I feel that it is not an obligation, but something that comes from God.

I: You mentioned that when you provide support you have a “Christian” moment (…)

P: Yes, I don’t impose my religion on them, but I always say…and it is always my saying…one of the phrases that I use the most [with the patients] is, “God is in control, have peace.”

I: Have you seen something positive [as a result]?

P: Yes, they do. They say, “thank you, amen”…and that word is usually used by people who were converted or were in the Church, because not everyone uses “amen.” Then you realize that they are receiving, even a little glimpse, of knowing that God is aware of them.

I: How does religion impact your daily life and nursing profession?

P: As a professional, in the past, I tried not to link religion with my profession, in the sense that I wanted to respect the space of each patient’s beliefs. But at present, I think [religion] can influence positively in the sense that if you try to bring a positive message, a better view of things, the patient might accept it in a positive way. As a professional, I understand that religion can have a positive impact on the lives of HIV patients who are going through that process of denial and acceptance of their condition.

Another participant explained why she talked with her patients about the love of God and his compassion toward PWHA during health care delivery:

I: When you say that you talk about the love of God, what do you mean?

P: They are people who are already aware of everything they have done. Most of them have been with the condition for a short time and are reluctant and in denial…but the persons who are already in treatment for 2 or 3 years, they know that they have to think about more positive things, and that is what brings them closer to God. That would be thinking of the Lord, and his love, that still has them alive.

Finally, another participant described the importance of religious beliefs in her life and explained why she tries to speak with her patients about God’s love and how Jesus will accept them:

I: How could religion impact the provision of health services by nurses?

P: I have my religious beliefs and I am a Christian woman. I have my moral values that are very different, perhaps from those who raised me…but I try to tell them [patients] that there is a God who loves you as I love you. As I accept them, Jesus accepts them. But I cannot use my religion to persecute them or to tell them you will be punished. If you follow this path God will punish you…

The 2 previous categories have shown that religion not only plays an important part in participants’ lives but that they integrate it into their health care delivery practices. In our final category, we examine how religion can foster highly stigmatizing notions regarding HIV.

### Religion and HIV-Related Stigma

In the prior section, we cited the following phrase used by a nurse in our study: “They are people who are already aware of everything they have done.” Such phrases may have a subtext of stigma in that they implicitly blame the PWHA for his/her infection. Our analysis revealed more explicit expressions of this linkage between religion and blame. In this section, we included narratives in which participants used their religious beliefs to explain a patient’s HIV infection. Some of them understood that HIV infection was the outcome of “sinful” behaviors that are condemned in the Bible (according to these participants). Some participants also believed that HIV infection was a consequence of one’s disrespect for the body resulting from the disobedience of God’s Commandments (eg, using drugs, being a homosexual person, and/or having multiple sexual partners). One nurse verbalized this link between religion and HIV-related stigma in the following manner:

I: Tell me about the impact of religion on the HIV/AIDS epidemic?

P: I come from a church that we call the Seventh-day Adventist. Our foundation is in God’s law. To fulfill God’s law, in our belief system, we maintain a clean body from alcoholic beverages, we don’t smoke, and we eat healthy. Many health conditions come from the bad treatment that you have given to the body. In the Ten Commandments, there are many things that you must fulfill, [for example] don’t commit adultery. I say, well I have my wife and she is the only one with whom I must be. God says that by looking at that other woman, you have committed adultery. So, when we see these epidemics, [and ask] why are so many dying? If there is disobedience, you are going to fall into that (HIV).

Participants mentioned disobedience of religious norms as the source of the HIV epidemic. They also described religion as a way to avoid sinful behaviors that could lead to HIV infection. For example, one participant described her interaction with a patient and how religion had rid him of his desire for men, which was interpreted as a catalyst for HIV infection. She stated:

P: …when he realized that he had HIV, he started to cry, and he said, “God I realize that I was making a life that is not what you wanted. Take away my desire for men. From now on, you take away the desire and I promise that I will not fall again.” It has been 18 years and he has no relationships with men or women. OK! He says “look at me, I thank God every day because I can see the most handsome man and for me there is no carnal desire”

Participants were aware that social stigma surrounded PWHA and that it could impact the services they receive. One participant described an experience where other nurses denied care to PWHA due their religious beliefs and practices:

I: I would like to know more about how religion impacts the nursing profession.

P: I have experiences with colleagues who are of a specific religion and they refuse to intervene with this type of patient [PWHA], because God doesn’t allow it, or because it goes against their religious beliefs. Even though they [nurses] receive an orientation, because you cannot decide who you offer a service to or not. You are a professional and you have to treat everyone the same. Still, there are people [nurses] that say to you, “I am of this religion and I cannot intervene with that person [PWHA] because it goes against my person and my church. I cannot interact with the patient.” Not even a verbal interaction as a health professional with the patient.

Finally, a nurse described how HIV was linked to individual characteristics that increased their risk for infection. These characteristics overlapped with religious beliefs that participants associated with sinful behaviors such as having multiple sexual partners and drug use. A nurse mentioned the following:

P: (People) who have sex with everybody…people that you see go out a lot with different people who are having sex with everyone. Drug users too…You go to have a drink and that night you meet a girl and you think that she is the most beautiful and most precious because you are drunk. The next day she is a person from the streets, a prostitute, and then she tells you “Welcome to the AIDS club.”

I: Do you think that having multiple partners is a common practice?

P: Yes, I understand that you don’t have that sense of caring for yourself as a person. Because if you are with several persons and you don’t protect yourself, you know…as they say, we can see their faces but don’t know their hearts. You know, people may not look sick, but inside we don’t know what they have.

## Discussion

This study aimed to explore the role of religion on HIV-related stigma among Puerto Ricans nurses. To our knowledge, this is the first study to explore the role of religion and HIV-related stigma among Puerto Rican nurses who were providing services to PWHA at the time the study took place. The results from our qualitative interviews revealed a concerning scenario for HIV-related stigmatization by nurses, as expressions of stigma were embedded in a larger cultural framework intimately linked to religious beliefs and practices.

Nurses in our study described their religious beliefs and practices as an intrinsic part of their daily lives. Participants described the social and personal significance of engaging in religious activities, as these were culturally important for their lives and communities. Religion had been a crucial part of their childhood and was still an important source of comfort in their adulthood. Moreover, their religion influenced their career path as nurses and was an important part of their professional identity development. Their religious outlook on their nursing practice seemed to evoke positive descriptions of this relationship. It fostered a desire to help and be of service to others, which is an integral part of being a nurse. In this sense, results highlight the need to understand the interaction between nurses, patients, and their social-cultural context. In health care settings, nurses’ religious beliefs seemed to foster rapport with patients and strengthened their commitment to patients’ health. In light of this positive outlook, most participants understood that the inclusion of religion in their professional practice with PWHA had positive outcomes. Although these findings are important and echo other studies,^30,35-37^ the relationship between religion and the provision of nursing services for PWHA was more complex.

In some narratives, nurses described specific practices fostered by their religious beliefs that tended to blame PWHA for their HIV status. Infection with HIV was understood to be a consequence of personal characteristics and/or behaviors that were sinful or immoral (eg, drug use, homosexuality). Such beliefs hinder crucial aspects of HIV care, such as the promotion of healthy behaviors and emotional well-being among PWHA, timely access to treatment in stigma-free environments, and adherence to treatment.^18,27^ Moreover, our results shows the importance of understanding how religion could shape the way nurses provide services to PWHA, since in certain contexts their personal and religious beliefs may support quality health care delivery, while in others it could foster HIV-related stigmatization during the clinical encounter. Our findings emphasize the need to develop targeted stigma reduction interventions for Puerto Rican nurses that can reduce stigmatizing experienced by PWHA while affirming the positive influence of religiosity on nurses’ work. For such interventions to be developed, further research is needed to quantitatively measure how religion influences specific health care delivery outcomes. Considering the complexity of such an intervention—which would necessarily need to address deeply engrained cultural beliefs and their effects on different moments of the clinical encounter—future studies should include the use of innovative instruments and research designs to measure the impact of HIV-stigma on nurse–patient interactions. We envision the potential use of patient case descriptions, such as those we used in our qualitative interviews, to elicit and reflect on stigmatizing scenarios among nurses as a point of entrée into a potential stigma reduction intervention in this context.

This study had some limitations that should be noted. Most interviews were conducted in the nurses’ work settings, which could have had an impact on social desirability. However, our team took precautions to identify a private room in the work facility and guaranteed participants’ confidentiality. In addition, even though participants self-identified as Christians from different denominations, we did not explore variations among them that could influence HIV-related stigma manifestations. Future studies should explore nurses’ specific religious backgrounds and how these might influence stigma and its behavioral manifestations. In addition, analyses did not consider participants’ gender, sexual orientation, and amount of experience working with PWHA. These are factors that may influence health care delivery and should be quantitatively explored in subsequent research.
